# Therapeutic targeting of myeloid-derived suppressor cells involves a novel mechanism mediated by clusterin

**DOI:** 10.1038/srep29521

**Published:** 2016-07-13

**Authors:** Junmin Zhou, Sarah S. Donatelli, Danielle L. Gilvary, Melba M. Tejera, Erika A. Eksioglu, Xianghong Chen, Domenico Coppola, Sheng Wei, Julie Y. Djeu

**Affiliations:** 1 Department of Immunology, H. Lee Moffitt Cancer Center, Tampa, FL 33612, USA; 2 Department of Pathology, H. Lee Moffitt Cancer Center, Tampa, FL 33612, USA.

## Abstract

Myeloid-derived suppressor cells (MDSCs) constitute a key checkpoint that impedes tumor immunity against cancer. Chemotherapeutic intervention of MDSCs has gained ground as a strategy for cancer therapy but its mechanism remains obscure.We report here a unique mechanism by which monocytic (M)-MDSCs are spared, allowing them to polarize towards M1 macrophages for reactivation of immunity against breast cancer. We first demonstrated that curcumin, like docetaxel (DTX), can selectively target CD11b^+^Ly6G^+^Ly6C^low^ granulocytic (G)-MDSCs, sparing CD11b^+^Ly6G^−^Ly6C^high^ M-MDSCs, with reduced tumor burden in 4T1-Neu tumor-bearing mice. Curcumin treatment polarized surviving M-MDSCs toward CCR7^+^ Dectin-1^−^M1 cells, accompanied by IFN-γ production and cytolytic function in T cells. Selective M-MDSC chemoresistence to curcumin and DTX was mediated by secretory/cytoplasmic clusterin (sCLU). sCLU functions by trapping Bax from mitochondrial translocation, preventing the apoptotic cascade. Importantly, sCLU was only found in M-MDSCs but not in G-MDSCs. Knockdown of sCLU in M-MDSCs and RAW264.7 macrophages was found to reverse their natural chemoresistance. Clinically, breast cancer patients possess sCLU expression only in mature CD68^+^ macrophages but not in immature CD33^+^ immunosuppressive myeloid cells infiltrating the tumors. We thus made the seminal discovery that sCLU expression in M-MDSCs accounts for positive immunomodulation by chemotherapeutic agents.

Breast cancer is the leading cause of cancer among women^1^. Chemotherapeutic targeting of tumor cells has been the standard of care but can be limited by development of chemoresistance. Thus, there is a need to mobilize the immune system against cancer, especially because of its specificity and ability to recognize chemo-resistant tumor cells as well as its long lasting T cell memory that can prevent tumor recurrence or metastasis. Immunotherapy and vaccine studies using antigens associated with breast cancer such as Her2^2^,^3^, however, face critical barriers related to an immunosuppressive environment linked to the induction of inhibitory myeloid lineage cells^4^,^5^,^6^,^7^,^8^. In the Her2^+^ 4T1 murine breast tumor model we find two distinct subsets of immature myeloid-derived suppressor cells (MDSCs) based on Ly6G or Ly6C expression, i.e., CD11b^+^Ly6G^+^Ly6C^low^ granulocytic (G)-MDSCs and CD11b^+^Ly6G^−^Ly6C^high^ monocytic (M)-MDSCs, in the spleen as the tumor progresses^9^. Similar results have been observed by others^10^,^11^. Mature tumor-associated macrophages of M1 and M2 phenotypes also develop in tumor bearers^4^,^5^,^6^,^7^,^8^,^12^. M-MDSCs can differentiate into mature M1 macrophages, which in turn trigger anti-tumor T cell responses^5^. On the other hand, G-MDSCs and M2 cells are highly immunosuppressive and they constitute the majority of the cells in tumor bearers^13^. Earlier, we reported a novel finding that docetaxel (DTX) selectively disrupts G-MDSCs and M2 cells while sparing M-MDSCs and expanding M1 cells, resulting in significant antitumor immunity and reduced tumor burden^9^. Others have reported similar findings with numerous chemotherapeutic compounds that target STAT3, tyrosine kinases or PDE5, as well as ATRA, paclitaxel, gemcitabine and 5-fluorouracil^14^,^15^,^16^,^17^,^18^,^19^,^20^.

The mechanism of sensitivity to multiple unrelated drugs and the disparity in drug sensitivity between G-MDSCs and M-MDSCs and between M1 and M2 macrophages is unknown. There must be intrinsic differences in these cells that allow for this effect. We previously documented that DTX and TRAIL resistance of tumor cells is due to expression of secretory/cytoplasmic clusterin (sCLU)^21^,^22^. Clusterin has been widely-associated with chemoresistance and cancer progression^23^. It is detected in most solid tumors, particularly in high grades and advanced stage of disease, in prostate, renal, bladder, breast, ovarian, colon, cervical, pancreatic carcinoma, hepatocarcinoma as well as osteosarcoma, melanoma and lymphoma^23^,^24^,^25^,^26^. sCLU is cytoprotective against a wide range of chemotherapeutic agents including paclitaxel, cisplatin, doxorubicin, etoposide, gemcitabine, Ara-C and carboplatin^23^,^25^,^27^. We recently made the seminal discovery that development of drug resistance may be due to a specific crosstalk between dying and remnant live tumor cells^27^. We demonstrated that dying tumor cells under DTX treatment release HMGB1 as a danger signal that binds TLR and RAGE on the neighboring live remnant tumor cells to trigger sCLU induction. Acquistion of sCLU allows them to resist apoptosis and begin expansion into a drug resistant clone. sCLU works by binding Bax, preventing its entry into mitochondria to release cytochrome c and activate caspases^27^. Despite the well-accepted presence of sCLU in tumor cells, its expression in immune cells has to date not been reported.

Recently, curcumin derived from plant, Curcuma longo, has shown efficacy in the treatment of breast cancer^28^,^29^. Curcumin is known for its high anti-oxidant capacity and lack of *in vivo* toxicity in humans treated for various inflammatory diseases as well as cancer^30^. Curcumin’s anti-inflammatory activity is mediated by inhibition of NF-κB and NF-κB -regulated genes^31^. In cancer, curcumin inhibits tumor cell proliferation and induces apoptosis by interfering with multiple molecular targets including signal cascades linked to NF-κB and STAT3^32^,^33^. Moreover, it enhances interferon-gamma (IFN-γ) production and CD8 cytotoxic function against tumor cells *in vivo*^34^.

In this study, we utilized the Her2^+^ 4T1 mammary carcinoma model to analyze the effect of curcumin on MDSCs and assess its potential therapeutic value. Like DTX, we found that curcumin drastically reduced the number of G-MDSCs while sparing M-MDSCs in tumor bearers. To account for this differential sensitivity to curcumin and DTX, examination of sCLU expression in M-MDSCs and G-MDSCs followed by analysis of cell death indicated that selective sCLU expression in M-MDSCs protected them from DTX or curcumin toxicity, leaving G-MDSCs vulnerable to Bax-mediated apoptosis, resulting in loss of the immunosuppressive myeloid phenotype and recovery of T cell function.

## Results

### Curcumin reduces tumor burden and proportion of MDSCs *in vivo*

**Figure 1:**
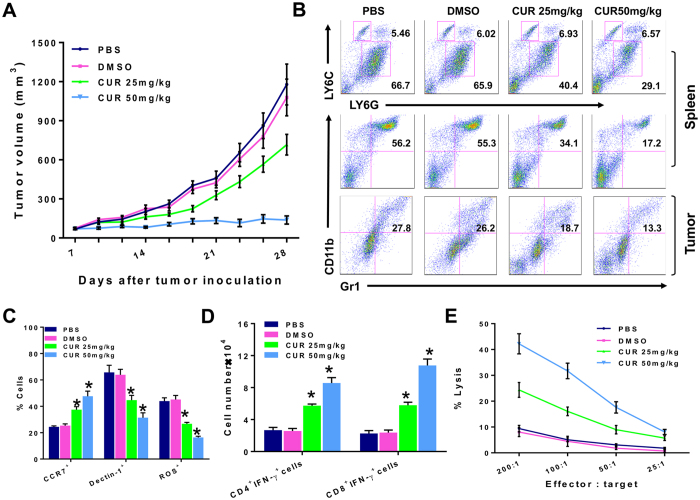
Curcumin reduces tumor burden and proportion of MDSCs with upregulation of T cell immunity *in vivo*. (**A**) BALB/c mice (n = 5/group) bearing 7-day subcutaneous 4T1 mammary carcinoma, 4–6 mm in diameter, were injected intraperitoneally with 25 or 50 mg/kg of curcumin (CUR), DMSO control or PBS three times weekly for three weeks and tumor size was evaluated with vernier calipers every 2–3 days. Experiments were performed at least three times. Data shown are the mean tumor volumes (mm^3^) ± standard error (SE). (**B**) Spleen cells or tumor-infiltrating mononuclear cells pooled from each group of mice at day 28 were analyzed by flow cytometry. The middle panel and bottom panel show the percentage of Gr1^+^CD11b^+^ (MDSCs) cells and the top panel shows the percentage of monocytic (M)-MDSCs (Ly6C^hi^Ly6G^lo^w) and granulocytic (G)-MDSCs (Ly6C^lo^Ly6G^hi^) cells among the Gr1^+^ cells. (**C**) Pooled splenocytes from each group at day 28 were stained with Gr1-PE, CD11b-APC, CCR7-FITC or stained with Gr1-PE, CD11b-APC, Dectin-1-FITC to assess for CCR7 and Dectin-1 expression of MDSCs by flow cytometry. Data represents the mean percentages ± SE of CCR7^+^Gr1^+^CD11b^+^ MDSCs and Dectin-1^+^Gr1^+^CD11b^+^ MDSCs from three independent experiments (*P < 0.05). To assess ROS production, another set of splenocytes were cultured with 3 μM dichlorodihydrofluorescein diacetate (DCFDA) followed by staining with antibodies to CD11b and Gr1, and analyzed by flow cytometry. Data shown are gated on CD11b^+^Gr1^+^ cells. (**D**) Splenocytes from each group at day 28 were stained with antibodies to CD8 or CD4 and IFN-γ. Data shown are the mean cell number ± SE of IFN-γ-positive CD8^+^ or CD4^+^ cells (*P < 0.05). (**E**) Splenocytes from each group at day 28 were plated at varying ratios with ^51^Cr-labeled 4T1 tumor targets. After 18 h at 37 °C, supernatants were harvested and the percentage of specific ^51^Cr release was determined by the following equation: (experimental cpm − spontaneous cpm)/(total cpm incorporated − spontaneous cpm) × 100. All determinations were done in triplicate, and the SE of was calculated and was typically 5% of the mean or less. All *in vitro* data are from a representative experiment of 3 experiments performed.

To examine the *in vivo* antitumor effect of curcumin, BALB/c mice bearing 7 day established subcutaneous 4T1 mammary tumors, between 4–6 mm in diameter, were injected intraperitoneally with curcumin or DMSO control and followed for tumor growth up to day 28. We observed that curcumin at 50 mg/kg almost completely retarded tumor progression as compared to DMSO-treated tumor-bearing mice, and the effect was dose-dependent with 25 mg/kg curcumin having a lesser effect (Fig. 1A).

In 4T1 tumor-bearers, Gr1^+^CD11b^+^ MDSCs robustly expand in the spleen^9^,^13^,^35^. To assess the effect of curcumin on MDSCs *in vivo*, we conducted a flow cytometric analysis of Gr1^+^CD11b^+^ splenocytes from 4T1 tumor-bearers treated with curcumin or DMSO control. By day 28 post tumor injection, Gr1^+^CD11b^+^ MDSCs constituted 55.3% of the spleen cells in DMSO-treated tumor-bearers, whereas the 25 and 50 mg/kg curcumin treatments reduced the proportion of MDSCs to 34.1% and 17.2%, respectively (Fig. 1B, middle panel). Interestingly, this reduction was observed only in the CD11b^+^Ly6G^+^Ly6C^low^ G-MDSC subset but not in the CD11b^+^Ly6G^−^Ly6C^high^ M-MDSC subset (Fig. 1B, top panel). Ly6G^+^ G-MDSCs cells were reduced from over 65.9% in DMSO-treated mice to 40.4% and 29.1% in the spleens of 25 mg/kg and 50 mg/kg curcumin-treated tumor bearers respectively, while the percentage of Ly6C^+^ M-MDSCs remained unchanged despite treatment, being maintained around 6.02% to 6.57%. It is also important to determine if intratumoral MDSCs are affected by curcumin administration in tumor bearers. Analysis of tumor-infiltrating myeloid cells showed the presence of 27.8% Gr1^+^CD11b^+^ MDSCs in day 28 established tumors in mice (Fig. 1B, bottom panel). While DMSO treatment did not affect the level of Gr1^+^CD11b^+^ MDSCs, curcumin treatments at 25 and 50 mg/kg markedly reduced the proportion of intratumoral MDSCs to 18.7% and 13.3%, respectively.

Moreover, the percentage of CCR7^+^ MDSCs (M1-phenotype) was significantly increased, with concomitant decrease in Dectin-1^+^ MDSCs (M2-phenotype)^36^ (Fig. 1C). MDSCs can mediate T cell suppression via secretion of potent factors, including arginase, nitric oxide (NO), and reactive oxygen species (ROS)^4^,^37^,^38^. As such, we investigated whether these factors were affected by curcumin treatment. We found that curcumin had no effect on arginase or NO production in MDSCs (data not shown), but ROS production was significantly reduced (Fig. 1C).

We next examined if loss of G-MDSCs relate to enhanced T cell function in curcumin-treated mice. Splenocytes from curcumin-treated tumor bearers had more CD4 and CD8 T cells expressing IFN-γ than did DMSO-treated mice (Fig. 1D), suggesting a promotion of proinflammatory adaptive responses by curcumin. We also found that splenic CD3^+^ T cells purified from curcumin-treated tumor bearers had substantially higher cytotoxicity against ^51^Cr-labelled 4T1 tumor cells than T cells from DMSO-treated tumor bearers (P < 0.01; Fig. 1E). These data suggest that curcumin treatment abrogated immunosuppression by MDSCs leading to potent cytotoxic T cell responses against the tumor.

**Figure 2:**
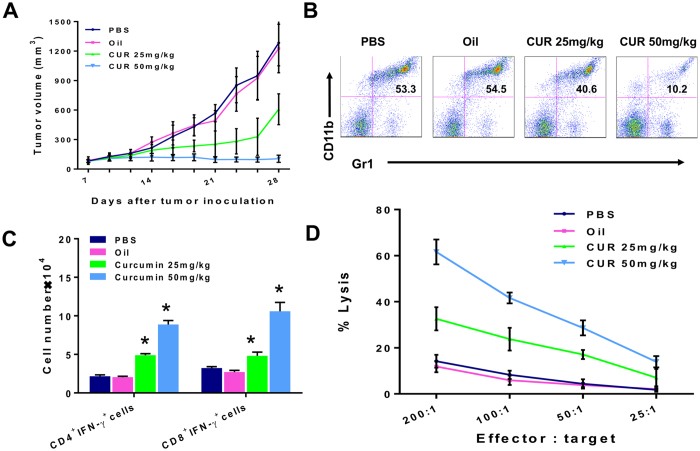
Oral Curcumin suppresses MDSCs and enhances T cell immunity, resulting in tumor inhibition in 4T1 tumor bearers. 4T1 mammary carcinoma cells (5 × 10^5^/mouse) were injected subcutaneously in BALB/c mice (n = 5/group). At seven days post injection, the mice were fed 25 or 50 mg/kg of curcumin mixed with piperine at a 20:1 (mg/kg:mg/kg) in olive oil, control olive oil or PBS orally (by gavage) three times weekly for three weeks. Tumor size was manually measured every 2–3 days with vernier calipers. (**B**–**D**) Spleen cells pooled from each experimental group of mice at day 28 were stained with antibodies against Gr1 and CD11b (**B**), CD4, CD8 and IFNγ (**C**) for flow cytometric analysis or tested for cytoxicity against ^51^Cr-labeled 4T1 tumor targets (**D**). All *in vitro* data are from a representative experiment of 3 experiments performed.

More importantly, oral administration of curcumin, which is the preferred route in man, in 4T1 tumor-bearering mice had similar efficacy in reducing tumor burden (Fig. 2A). Oral gavage with curcumin mixed in olive oil at 25 mg/kg and 50 mg/kg in 7-day tumor bearers showed tumor inhibition in a dose dependent manner while the olive oil itself had no effect. Reduction in splenic Gr1^+^CD11b^+^ MDSCs accompanied tumor suppression in a dose-dependent manner. From 53.3% and 54.5% Gr1^+^CD11b^+^ MDSCs in tumor bearers treated with PBS control and olive-oil control respectively, oral curcumin at 25 mg/kg and 50 mg/kg brought these levels down to 40.6% and 10.2% respectively (Fig. 2B). With the loss of MDSCs, splenic CD4^+^IFNγ^+^ cells and CD8^+^IFNγ^+^ cells were accordingly increased (Fig. 2C) accompanied by recovery of lysis against 4T1 tumor cells in curcumin-fed tumor bearers (Fig. 2D).

### Clusterin is responsible for the selective survival of Ly6C^+^ M-MDSCs

**Figure 3:**
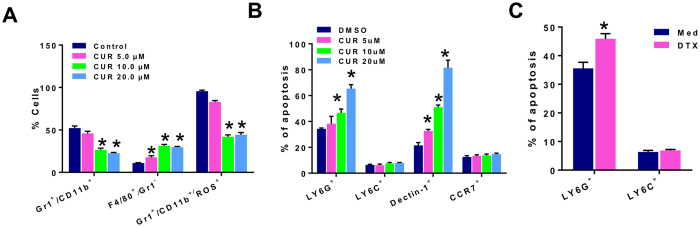
Curcumin reduces MDSCs *in vitro* by inducing apoptosis in Ly6G^+^ G-MDSCs and Dectin-1^+^ M2 cells but not Ly6C^+^ M-MDSCs or CCR7^+^ M1 cells. (**A**) Spleens pooled from 5 mice bearing 2 week-old 4T1 tumors were harvested, and Gr1^+^ cells were purified by positive magnetic selection. Purified MDSCs (>90% Gr1^+^CD11b^+^) were incubated for 72 h in the presence of curcumin or DMSO and phenotypic subsets wereassessed for presence of Gr1, CD11b, F4/80 or ROS by flow cytometry. (**B**) Another set of MDSCs pretreated with curcumin or DMSO were analyzed for apoptosis by AnnexinV^+^ staining in Ly6G^+^ G-MDSC, Ly6C^+^ M-MDSC, CCR7^+^ M1 and Dectin-1^+^ M2 cells (**C**) Purified MDSCs from 2-week old tumor bearers were incubated with 10 ng/ml of DTX for 24 h and then analyzed for apoptosis byAnnexinV^+^ staining in Ly6G^+^ and Ly6C^+^ MDSCs. Bar graphs show mean ± SE of three independent experiments. *P < 0.05.

To examine if curcumin directly affected MDSCs, we purified Gr1^+^ MDSCs from spleens of 4T1 tumor bearers and analyzed the phenotypes of the MDSCs remaining after *in vitro* incubation with curcumin or control DMSO for 72 h. Culture was conducted in 4T1 tumor cell conditioned medium (TCCM) with low dose GMCSF to simulate the tumor microenvironment and maintain MDSCs. Flow cytometric analysis indicated that curcumin at 5, 10, 20 μM, reduced the percentage of Gr1^+^CD11b^+^ MDSCs in a dose dependent manner (Fig. 3A). Remarkably among these cells, the Gr1^−^F4/80^+^ cells were increased, suggesting the differentiation towards M1 macrophages while Gr1^+^CD11b^+^ ROS^+^ cells associated with immune suppression were decreased. Because curcumin is known to induce apoptosis in many tumor cell types^29^,^32^,^33^, we queried whether the change in phenotypes in MDSCs treated with curcumin could be accounted for by apoptosis. Analysis by Annexin V staining indicated that only the Ly6G^+^ subset but not the Ly6C^+^ subset showed an increased percentage of apoptosis upon curcumin treatment for 24 h (Fig. 3B). Additionally, curcumin induced apoptosis in Dectin-1^+^ cells (M2 phenotype) in a dose-dependent manner without killing CCR7^+^ cells (M1 phenotype) in splenic MDSCs from tumor bearers (Fig. 3B). Lastly, *in vitro* treatment of MDSCs with another chemotherapeutic agent, DTX, produced a similar effect as curcumin, inducing apoptosis in Ly6G^+^ MDSCs but not Ly6C^+^ MDSCs (Fig. 3C).

**Figure 4:**
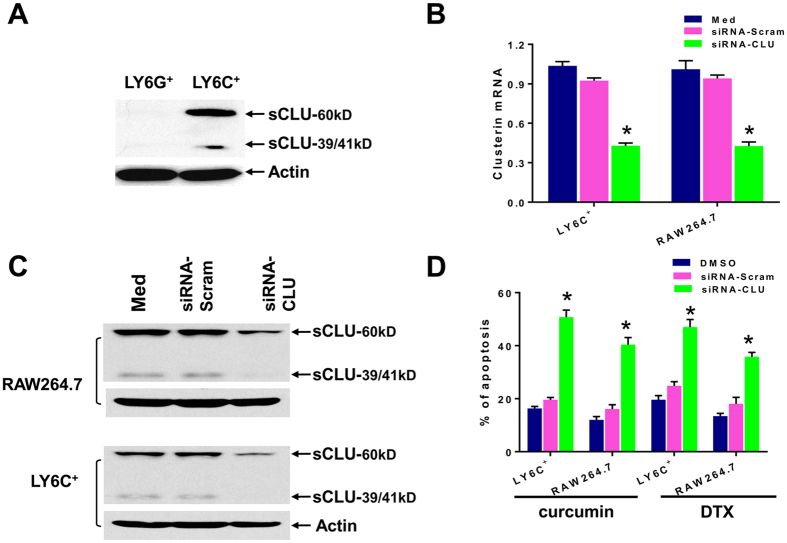
Clusterin mediates selective survival of Ly6C^+^ MDSCs. (**A**) Western blot analysis of secretory/cytoplasmic clusterin (sCLU) in Ly6G^+^ G-MDSC and Ly6C^+^ M-MDSC isolated from tumor bearers. Both the 60 kD and the 39/41 kD forms can be detected in Ly6C^+^ but not Ly6G^+^ cells. Actin was used as loading control. (**B**,**C**) Ly6C^+^ M-MDSC or RAW264.7 macrophages were transfected with antisense-scramble or antisense-CLU for 24 h, and then tranfection efficiency was checked by analysis of sCLU expression by Q-PCR (**B**) and western blot (**C**) analysis. (**D**) Analysis of apoptosis by AnnexinV^+^ staining indicated that knockdown of sCLU by siRNA-CLU induced apoptosis in both RAW264.7 cells and M-MDSCs treated with 20 uM curcumin or 10 ng/ml DTX. Bar graphs show mean ± SE of three independent experiments. *P < 0.05.

Based on the wide-association of sCLU with chemoresistance in tumor cells, we set out to determine if sCLU also operate preferentially in M-MDSCs to provide survival advantage. Indeed, western blot analysis indicated that sCLU was present only in Ly6C^+^ M-MDSCs but not in Ly6G^+^ G-MDSCs (Fig. 4A). We detected the unspliced cytoplasmic 60 kD form which is consistently reported to confer survival advantages to tumor cells^23^,^27^. We also detected a small amount of the 39 kD α and 41kD β chains formed from splicing and glycosylation of the 60 kD protein to become the mature secreted heterodimer^23^,^27^. To confirm that sCLU is the key anti-apoptotic protein in M-MDSCs, we transduced antisense sCLU into M-MDSCs from tumor bearers, with scrambled siRNA as the control and checked that sCLU expression was accordingly downregulated at the mRNA and protein levels by Q-PCR and western blot analysis respectively (Fig. 4B,C). We also included the RAW264.7 cell line which constitutively express sCLU as another source of macrophages. RAW264.7 cells have the phenotype of Gr1^−^ CD11b^+^ F4/80^+^ MHC-II^+^ CCR7^+^ M1 macrophages (Fig. 1S). Under these conditions, both curcumin and DTX could now markedly promote apoptosis in antisense CLU-silenced Ly6C^+^ and RAW264.7 macrophages, as compared to scrambled siRNA-control (Fig. 4D). Curcumin alone induced only 19.6% apoptosis in M-MDSCs or 16.1% apoptosis in RAW264.7 cells; yet after sCLU silencing, curcumin induced 50.8% apoptosis in M-MDSCs and 40.4% apoptosis in RAW264.7 cells. Similar results were obtained with DTX treatment, indicating that sCLU is cytoprotective against unrelated toxic agents (Fig. 4D).

### Knockdown of clusterin increases apoptosis via impeding Bax translocation into mitochondria

**Figure 5:**
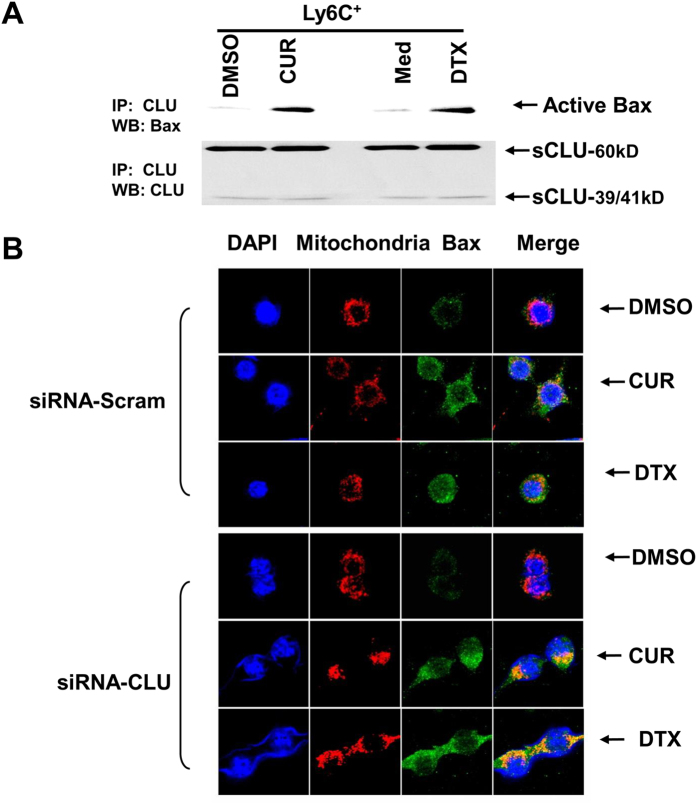
Clusterin prevents apoptosis by inhibiting Bax translocation to mitochondria (**A**) sCLU immunoprecipitated from Ly6C^+^ MDSCs treated or untreated 12 h with 20 uM curcumin (CUR) or 10 ng/ml DTX were subjected to western blot analysis with 6A7 anti-active Bax antibody. sCLU did not bind active Bax in control DMSO-treated Ly6C^+^ cells but did so after curcumin or DTX treatment. (**B**) RAW264.7 macrophages were transfected with antisense-scramble or CLU for 24 h and then treated with DMSO, 20 μM curcumin or 10 ng/ml DTX for 2 h. Cells were incubated with 125 nM MitoTracker Red CMXRos for 30 min at 37 °C (red fluorescence), then stained with anti-6A7 followed by Alexa Fluor 488-conjugated antibody (green fluorescence). Cells were examined by confocal microscopy (magnification, ×630). Active Bax shows little translocation to mitochondria in scrambled control but almost complete colocalization in anti-sense CLU-transfected macrophages upon curcumin or DTX treatment. One representative set of images of three independent experiments is shown.

In tumor cells, it is reported that sCLU blocks apoptosis by interacting with activated, conformation-altered Bax, thereby preventing Bax from translocating into mitochondria and exerting its proapoptotic activity^39^. To explore if this process operates in M-MDSCs for cytoprotection, we first examined the ability of curcumin or DTX to activate Bax in Ly6C^+^ MDSCs and induce its association with sCLU. We therefore immmunoprecipitated sCLU from purified Ly6C^+^ MDSCs before and after 12 h treatment with either curcumin or DTX and western blotted the immunoprecipitates with a specific antibody that recognizes the activated conformation-altered form of Bax, 6A7. Such analysis indicated that active Bax was present in the sCLU immunoprecipitates of both curcumin and DTX-treated cells, but it could not be detected in untreated control cells (Fig. 5A). To confirm that sCLU binding of active Bax was responsible for loss of movement of Bax into mitochondria, we next depleted sCLU by transfection of antisense-CLU into RAW264.7 macrophages and investigated whether curcumin or DTX treatment can now mobilize Bax to the mitochondria to initiate the caspase cascade. Confocal microscopic analysis did not detect any active Bax (green) in DMSO control-treated macrophages (Fig. 5B, top panel). However, active Bax was induced after curcumin or DTX treatment in scrambled antisense control-transduced macrophages and displayed some but incomplete localization with Mitotracker Red-labeled mitochondria. Upon transduction with antisense-CLU, the macrophages now showed complete colocalization of active Bax with mitochondria, signaling apoptosis (Fig. 5B, bottom panel). Thus loss of sCLU allows active Bax movement to mitochondria, indicating that presence of sCLU in macrophages is critical for prevention of Bax-mediated apoptosis in drug-treated cells.

### Clusterin is overexpressed in CD68 mature macrophage cells of human breast cancer tissues

**Figure 6:**
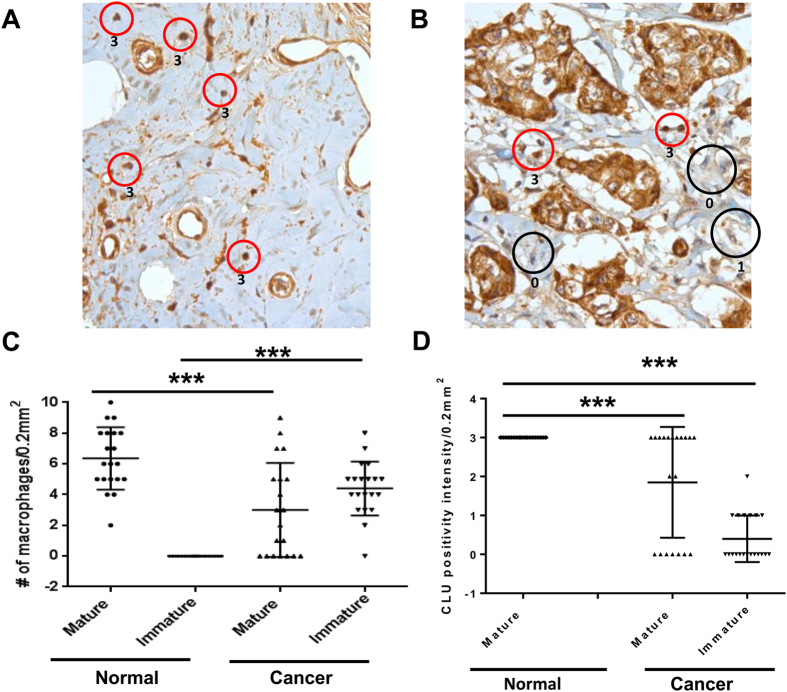
Clusterin is expressed in mature macrophages and not in immature myeloid cells in human breast tumor tissues from patients with invasive ductal carcinoma. Twenty formalin-fixed paraffin-embedded human normal breast tissues (**A**) and breast cancer samples (**B**) were stained for sCLU and the numbers of macrophages (red circle) and immature myeloid cells (black circle) were visually assessed by morphology. The intensity of sCLU staining was scored from 0 to 3, with 0 being the lowest, which is shown next to each circle. Note that the breast cancer cells are all sCLU positive, as has been reported by others. (**C**) The twenty samples from normal and breast cancer were then analyzed for the average distribution of mature and immature myeloid cells expressing sCLU and the total number of mature macrophages or immature macrophage cells per tissue sample is shown. (**D**) Intensity of sCLU staining for each macrophage or immature myeloid cells per tissue sample. P values (***P < 0.001) were generated by Student’s t test.

It has long been known that many leukocytes, including macrophages and immunosuppressive immature myeloid cells, are present in tumor stroma^4^,^5^,^40^. We thus evaluated their presence in tumor tissues from 20 breast cancer patients with invasive ductal carcinoma and compared them to 20 normal breast tissues from individuals undergoing breast reduction (Fig. 6). Histological examination of myeloid/macrophage morphology revealed that normal breast tissues contained primarily mature macrophages (red circle) and scoring the intensity of sCLU staining at a scale of 0 to 3 with 0 being the lowest, depicted that normal macrophages all expressed a high level of sCLU at a scale of 3 (Fig. 6A). On the other hand, breast tumor tissues express not only mature macrophages but also a high number of immature myeloid cells (black circle) and these immature myeloid cells express hardly any sCLU at a scale of 0–1 (Fig. 6B). We calculated the average expression of mature and immature myeloid cells in the 20 normal and tumor samples, and confirmed that normal breast tissues have an average of (6.4 ± 2.0) mature macrophages with no detectable immature myeloid cells (0 ± 0) (Fig. 6C). In contrast to normal tissues, breast cancer samples showed markedly reduced number of mature macrophages (3.0 ± 3.1) and a significant infiltration of immature myeloid cells (4.4 ± 1.8). Thus, the number of mature macrophage cells is significantly higher in normal breast tissue samples than in breast tumor tissues (p < 0.001), while immature macrophage cells accumulate preferentially in breast tumor tissues (p < 0.001). Further analysis of the intensity of sCLU staining in these tissues indicated that mature macrophages in normal breast tissues express significantly higher levels of sCLU (intensity score 3.0 ± 0) than in breast tumor tissue (intensity score 1.9 ± 1.4, p < 0.001) while immature myeloid cells, primarily seen in breast cancer tissues, express barely any sCLU in comparison to normal macrophages (intensity score 0.4 ± 0.6, p < 0.001) (Fig. 6D).

**Figure 7:**
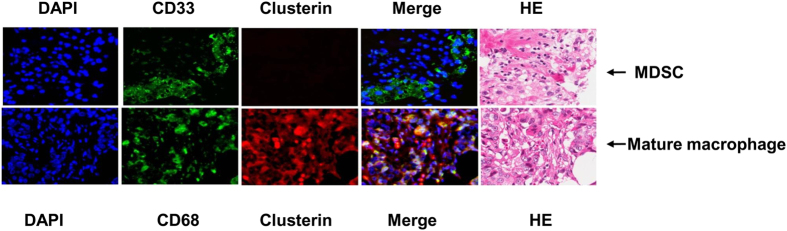
sCLU is expressed in CD68^+^ mature macrophages but not in CD33^+^ immature myeloid cells in invasive ductal carcinoma. Immunohistochemical fluorescent analysis of a paraffin-embedded section of invasive ductal carcinoma tissue was conducted by dual staining with primary rabbit anti-human CD68 and mouse anti–human sCLU, or rabbit anti-human CD33 and mouse anti-human sCLU, followed by anti-mouse Alexa-647 and anti-rabbit Alexa-488.

In man, immunosuppressive MDSCs are characterized as CD11b^+^CD14^−^CD33^+^ or HLA-DR^−^Lin^−^CD33^+^ cells^41^. On the other hand, mature macrophages are CD68^+^ 5. Using CD33 and CD68 as markers for MDSCs and mature macrophages respectively, we analyzed whether they infiltrate human breast cancer tissues and which population expresses sCLU. Dual-staining for sCLU and CD68 or CD33 indicated that sCLU was primarily expressed in CD68^+^ mature macrophages and not in immature CD33^+^ myeloid cells, as shown in a representative tumor tissue from a breast cancer patient in Fig. 7. Thus, human macrophages and immature myeloid cells in cancer are similar to those in mice bearing 4T1 tumors in expressing sCLU primarily in macrophages.

## Discussion

There is unrefuted evidence that checkpoint blockade can restore immunity to cancer. Such strategy has met high clinical success, especially with specific antibodies directed against inhibitory molecules on T cells such as PD1 and CTLA-4 in cancer patients^42^. Targeting immunosuppressive myeloid cells that develop in the tumor microenvironment in order to restore immune function is yet another pursuit that has gained momentum. Remarkably, several chemotherapeutic agents used traditionally to kill tumor cells in cancer patients as well as kinase inhibitors that target STAT3 and tyrosine kinases, have recently been found to be efficacious in altering the myeloid-mediated immunosuppressive environment, leading to adaptive T cell immunity and tumor regression^9^,^14^,^15^,^16^,^17^,^18^,^19^. Such chemotherapeutic agents exhibit unrelated mechanisms to target cancer cells and yet can eliminate MDSCs in tumor bearers, with expansion of M1-type macrophages that can enhance tumor immunity. We thus set out to identify the mechanism of chemoimmunodulation of the immune response in tumor bearers and made the novel discovery that sCLU is the common denominator for effective chemoimmunomodulation that restores adaptive immunity.

In this study, we first demonstrate that a chemopreventive agent, curcumin, long consumed for medical purposes with little toxicity in man^30^, is as effective as DTX, a standard anti-cancer agent previously reported by us^9^, in eliminating Gr1^+^CD11b^+^ MDSCs, whose immunosuppressive function is well documented. What is most remarkable is our finding that curcumin, like DTX, selectively eliminated splenic Ly6G^+^ G-MDSCs with preferential survival of Ly6C^+^ M-MDSCs, accompanied by an expansion of CCR7^+^F4/80^+^M1 but not Dectin-1^+^ M2 cells *in vivo* in mice bearing 4T1-Neu tumors. Such *in vivo* immune modulation by curcumin led to enhanced IFNγ^+^ CD4 and CD8 T cells with heightened capacity to lyse syngeneic 4T1 tumor cells, resulting in inhibition of tumor growth. We have earlier shown that the mechanism of T cell functional recovery in DTX-treated tumor bearers was directly due to the loss of the immunosuppressive function of MDSCs on T cells^9^. MDSCs taken from DTX-treated mice were unable to inhibit OT-II CD4 T cell proliferation in response to a specific OVA peptide. Although we did not perform the same experiments with curcumin, it is likely that curcumin also restored T cell function by eliminating the ability of MDSCs to suppress T cells. Most importantly, curcumin treatment *in vivo* was equally capable of reducing Gr1^+^CD11b^+^ MDSCs within the tumors (Fig. 1B), indicating its potency in modulating immunity in the tumor microenvironment.

Curcumin’s main action is via inhibiton of STAT3 and NF-κB^32^,^33^, which are exactly the same molecules identified in MDSCs for their immunosuppressive function^4^,^5^,^40^. In fact, STAT3 has been reported to control NAPDH oxidase involved in ROS production, which can disrupt macrophage maturation and T cell function^15^,^38^. Our observation of ROS depletion suggest that this pathway is functionally affected by curcumin in MDSCs. Curcumin reportedly is highly effective in suppressing Tregs^43^. In light of earlier reports that immature myeloid cells are responsible for development of Tregs in tumor bearers^44^, curcumin could affect this Treg pathway through direct action on MDSCs. Another important point is the equal effectiveness of oral versus subcutaneous administration of curcumin in immune reconstitution in 4T1 tumor bearers. Our positive results with oral curcumin contrasts with other studies which saw little benefit with the same route^30^,^45^. The failure in these studies was due to the poor absorption and bioavailablity of curcumin^46^, which can be corrected by addition of piperine isolated from black pepper^47^. Because we admix curcurmin with piperine for oral gavage, we were able to observe high potency in deleting MDSCs with resultant tumor regression, to the same extent achieved by intraperitoneal administration of pure curcumin. Taken together, curcumin may serve as a potent, non-toxic therapeutic agent that can be given orally to reactivate immunity against cancer. Indeed, clinical trials with curcumin have shown positive results that could in part be due to immune reactivation against the tumor^28^,^48^.

The manner of cell death of Ly6G^+^ G-MDSCs was by apoptosis. Because 2 unrelated agents, curcumin and DTX, exhibited the same selective toxicity on Ly6G^+^ G-MDSCs but not on Ly6C^+^ M-MDSCs, we examined if the anti-apoptotic protein, sCLU, long-associated with multi-drug resistance in tumor cells might be involved^23^,^24^,^25^,^26^. Indeed, we recently uncovered a specific interaction between dying and remnant live cells through a specific HMGB1/TLR4-RAGE pathway that induces sCLU in the live cells to develop chemoresistance in tumor cells^27^. Remarkably, sCLU was detected only in the cytoplasm of Ly6C^+^ M-MDSCs but not in Ly6G^+^ G-MDSCs. Proof of its role in selective survival of M-MDSCs was obtained with antisense sCLU overexpression in Ly6C^+^ M-MDSCs which abrogated their drug resistance properties under curcumin or DTX treatment. A macrophage cell line, RAW264.7, which constitutively expresses sCLU, was also rendered sensitive to curcumin and DTX upon transduction with antisense CLU. Thus, sCLU is active not only in tumor cells but also in myeloid cells to provide survival mechanisms and this property is especially critical for the preservation of antigen-presenting cells to maintain immunity against cancer.

Apoptosis is initiated by Bax activation and translocation to mitochrondria for activation of the caspase cascade. It is reported that sCLU in tumor cells acts by binding active Bax, impeding their translocation to mitochondria to initiate apoptotic caspase casade^39^. We found that this pathway is operative in Ly6C^+^ M-MDSCs. Notably, we detected Bax activation in Ly6C^+^ M-MDSCs upon curcumin or DTX treatment and it co-immunoprecipitated with sCLU. To confirm that sCLU binding of active Bax retards its mobility to mitochondria, we utilized an immunohistochemical approach to visualize active Bax movement within macrophages. Our results confirmed that active Bax in curcumin or DTX-treated macrophages remain distributed in the cytoplasm with little mobilization to mitochondria, thus preventing apoptosis. However, upon transduction with antisense-CLU, RAW264.7 macrophages can now colocalize active Bax with mitochondria, initiating the apoptotic cascade. Thus, sCLU acts as a Bax trap in macrophages as seen in tumor cells to resist cell death.

To understand if sCLU might be involved in human cancer, we examined tumor-infiltrating myeloid cells in 20 breast cancer patients with invasive ductal carcinoma. As compared to normal breast tissues, we found that breast tumor biopsies displayed a heightened accumulation of immature myeloid cells with much less mature macrophages. Most importantly, sCLU was detected only in mature macrophages in normal and breast tumor tissues, but not in immature myeloid cells that dominate in tumors. In other studies, immature immunosuppressive myeloid cells, identifiable by CD33 staining, are generally associated with a poor clinical outcome while CD68 staining to identify mature macrophages tends to correlate with better prognosis if aligned with HLA-DR costaining and not CD163 costaining^4^,^5^,^40^,^49^. Importantly, single CD68 staining of tumor tissues has been clearly associated with survival advantage in colorectal cancer and non-small cell lung carcinoma^50^,^51^. In order to detect sCLU in immature or mature macrophages, we costained sCLU either with CD33 or CD68. Our observation of selective expression of sCLU in infiltrating CD68^+^ macrophages but not in CD33^+^ immature myeloid cells within breast cancer tissues is an important new finding that explains the immune-restorative capacity of certain anti-cancer drugs. Indeed, the property of DTX and curcumin to selectively eliminate immunosuppressive myeloid cells and recover T cell immunity may be behind the positive outcome seen with clinical trials in cancer patients with such combination therapy^28^. Moreover, their use in combination with immunotherapy have seen a significant benefit accompanied by enhanced adaptive immunity, possibly related to differential survival of sCLU expressing M-MDSCs and M1 macrophages. In murine tumor models, curcumin enhances vaccine therapy using either mage-b-expressing Listeria vaccine against 4T1 breast cancer^52^ or Tryp-2 vaccine against B16F10 melanoma^53^. DTX was also demonstrated to enhance dendritic cell-based vaccine against B16 melanoma^54^. In these studies, enhanced T cell immunity as well as decreased Tregs and MDSCs was observed, similar to our current results^52^,^53^,^54^. In man, clinical trials with DTX and gemcitabine has each been used to boost immunotherapy against cancer and it appears that the sequence of drug and vaccine delivery was critical for the clinical outcome. In the protocols that showed clinical benefit, DTX^55^,^56^ or gemcitabine^57^ was given prior to vaccine adminstration, suggesting that elimination of immunosuppressive myeloid cells needs to occur before the vaccine can stimulate an adaptive immune response.

Our results taken together points to sCLU as a critical mediator of positive immunomodulation by chemotherapeutic/chemopreventive agents and accounts for the differential survival of M-MDSCs that can expand towards mature M1 macrophages to mount an immune response.

## Methods

### Reagents

Curcumin, piperine, actin antibody and protein G magnetic beads were purchased from Sigma-Aldrich, Inc (St. Louis, MO), Dichlorodihydrofluorescein diacetate (DCFDA) was purchased from Molecular Probes (Eugene, OR). The following antibodies were purchased from eBiosciences (San Diego, CA): anti-mouse-Ly6G and Ly6C PE, anti-mouse-CD11b APC, anti-mouse F4/80 FITC, anti-mouse CCR7 APC, anti-mouse Ly6G FITC, anti-mouse CD4 FITC, anti-mouse CD8 PE, anti-mouse Ly6C PE, isotype control antibodies. Annexin V Apoptosis kit, Bax 6A7 antibody, anti-mouse-IFN-γ APC were obtained from BD Biosciences (San Jose, CA). Biotinylated anti-Gr1, anti-Ly6C, anti-Ly6G and streptavidin microbeads were purchased from Miltenyi Biotech (Auburn, CA). Anti-mouse Dectin-1 was purchased from AbD Serotec. Clusterin antibodies were purchased from Upstate (Millipore, Billerica, MA). Clusterin siRNA and Scrambled negative control were purchased from OriGene (Rockville, MD). MitoTracker Red CMXRos, Alexa 488 anti-mouse secondary antibody and Lipofectamine 2000 were purchased from Invitrogen (Grand Island, NY).

### Mice, tumor establishment, and curcumin administration

Female BALB/c mice at 6 to 8 weeks of age were purchased from the National Cancer Institute (Frederick, MD) and kept in pathogen-free conditions in the animal facility of Moffitt Cancer Center. All experiments were performed in accordance the Univeristy of South Florida IACUC with preapproved institutional protocols within the guidelines of the Animal Care and Use Committee. The mouse breast tumor cell line, 4T1-Neu, was grown in RPMI 1640 with 10% heat-inactivated fetal bovine serum with 100 units/ml penicillin, 100 μg/ml streptomycin, and 2 mM glutamine^9^. BALB/c mice were inoculated subcutaneously (s.c.) in the flank with 5 × 10^5^ 4T1 mammary carcinoma cells. Tumor growth was monitored with bidirectional tumor measurements using calipers every 2–3 days and tumor volume was calculated using the formula V = 0.4ab^2^ with “a” as the larger diameter and “b” as the smaller diameter. Curcumin, diluted in DMSO, was given at different doses via intraperitoneal (i.p.) injection or mixed with piperine 20:1 (mg/kg:mg/kg) in olive oil for oral gavage three times a week starting on day 7 after tumor inoculation until completion of the experiment. At the time of curcumin treatment, the tumors were usually 4–6 mm in diameter.

### Cell isolation and culture

Spleens were harvested under sterile conditions. Splenocytes pooled from five mice per group or from 4T1-Neu tumor bearers were prepared by lysing red blood cells using ACK lysing buffer. Briefly, 1 × 10^8^ splenocytes were resuspended in 0.9 ml of cold MACS buffer (0.5% BSA in PBS with 2 mM EDTA), incubated with 100 μl of biotinylated antibodies against Gr1, Ly6C or Ly6G (Miltenyi Biotec Auburn, CA) for 20 min at 4 °C. Cells were washed with cold MACS buffer to remove unbound Gr1, Ly6C or Ly6G antibodies, and then incubated with 100 μl of streptavidin microbeads for 15 min at 4 °C. The Gr1^+^, Ly6C^+^ or Ly6G^+^ cell population was isolated on a MACS column according to the manufacturer’s instructions (Miltenyi Biotec).

The purity of the Gr1^+^, Ly6C^+^ or Ly6G^+^ cell population was evaluated by flow cytometry and exceeded 90%. Purified Gr1^+^, Ly6C^+^ or Ly6G^+^ cells were maintained in RPMI 1640 containing 20% fetal bovine serum, 20% 4T1 tumor-conditioned medium and 10 ng/ml mouse GM-CSF to mimick the tumor microenvironment^58^. 4T1-Neu mammary tumor cells were maintained in 10% (vol/vol) FBS/RPMI supplemented with 1% P/S, 1% L-glutamine (Gibco). Murine macrophage RAW264.7 cells were maintained in 10% (vol/vol) FBS/DMEM supplemented with 1% P/S, 1% L-glutamine (Gibco).

### Quantitative RT-PCR

Cells were lysed in TRIzol (Invitrogen), and RNA was purified and converted to cDNA (iScript cDNA Synthesis kit; BioRad). Real-time PCR was performed with iScript Syber- Green Supermix (BioRad). Primer sequences are as follows: clusterin (F 5′-GATGATCCACCAGGCTCAACAG-3′/R 5′-ACACAGTGCGGTCATCTTCACC-3′) and GAPDH (F-5′-TTCACCACCATGGAGAAGGC-3′/R-5′-GGCATGGACTGTGGTCATGA-3′). Experimental genes were normalized to GAPDH. Relative fold changes in expression were determined by using the comparative cycle threshold method (2−ΔΔCT).

### Immunohistochemistry and immunofluorescence staining

Serial 4-μm-thick paraffin sections taken from each representative block of 20 cases of invasive ductal carcinoma of the breast and 20 cases of normal breast tissue acquired from individuals undergoing breast reduction (obtained in accordance with the Moffitt Cancer Center IRB Human Subjects Board) were immunohistochemically tested for sCLU expression using the primary rabbit polyclonal antibody for sCLU (ab69644, Abcam, Cambridge, MA) at a dilution of 1:100, for 2 h at room temperature with the Ventana automated immunostainer Discovery XT (Ventana Medical Systems, Tucson, AZ). As a negative control, we used non-immune mouse sera, omitting the sCLU antibody during the primary antibody incubation step. The slides were read by a certified pathologist and co-author (DC) in a blinded fashion and the CLU protein expression levels were measured using the Allred semiquantitative scoring system.

Another set of tissues was manually stained for dual CD33 and sCLU expression, or dual CD68 and sCLU expression. Briefly, slides (4-μm) were deparaffinized with xylene, rehydrated in water/ethanol washes and blocked with goat serum. Then the slides were incubated either with primary mouse anti-human sCLU (Upstate/Millipore, Billerica, MA) and rabbit anti-human CD33 (LifeSpan BioSciences, Seattle, WA) or with mouse anti-human sCLU and rabbit anti-human CD68 (LifeSpan BioSciences, Seattle, WA) overnight at 4 °C. Next day, slides were incubated with Alexa 568 anti-rabbit or Alexa 647 anti-mouse secondary antibody for 30 min. The slides were mounted with Everbrite mounting medium and DAPI (Biotium, Inc., Hayward, CA), and then analyzed with an automated Zeiss Observer Z.1 inverted microscope through a 10 × /0.3 NA and 20 × /0.5 NA objective with DAPI, Cy5, and Alexa 568 filter. Images were captured by using the AxioCam MRm3 CCD camera and Axiovision (Version 4.7; Carl Zeiss). An H&E-stained serial section of the tissues were assessed by a board-certified pathologist (DC).

To detect Bax expression in mitochondria, RAW 267.4 macrophages being grown on glass coverslips were incubated with 125 nM MitoTracker Red CMXRos for 30 min at 37 °C. These cells were fixed with ice-cold methanol for 15 min at −20 °C and permeabilized with 0.2% Triton X-100 in PBS for 10 min. Cells were blocked with goat serum for 30 min and then incubated with monoclonal anti-active Bax (6A7, 1:200) for 60 min at room temperature followed by Alexa 488 anti-mouse secondary antibody (1:1000) for 30 min. Coverslips were mounted onto slides using Everbrite mounting medium with DAPI and colocalization of Bax with mitochondria was assessed using a confocal microscope (Carl Zeiss MicroImaging, Inc., Thornwood, NY).

### ^51^Cr release cytotoxicity assay

A ^51^Cr-release assay was performed as previously described^9^. CD3^+^ T lymphocytes isolated from spleen cells, which were pooled from five tumor bearers treated with curcumin or DMSO control, served as effector cells. Briefly, 4T1-Neu tumor cells were labeled with 200 μCi of ^51^Cr (Amersham Corp, Louisville, CO) in 0.2 mL of medium at 37 °C in a 5% CO_2_ atmosphere for 1 h. The labeled 4T1-Neu tumor cells were washed three times and added to the effector cells in triplicate wells of 96-well round-bottomed microplates at the 200:1, 100:1, 50:1 and 25:1 E:T ratios. After 18 h incubation at 37 °C, supernatants were harvested and counted in a γ-counter. The percentage of specific ^51^Cr release was determined by the following equation: (experimental cpm − spontaneous cpm)/(total cpm incorporated − spontaneous cpm) × 100. All determinations were done in triplicate, and the SE of all assays was calculated and was typically 5% of the mean or less.

### Western blot analysis

For immunoblot, 30–50 μg of protein was separated by 10% SDS-polyacrylamide gel electrophoresis, transferred onto polyvinylidene fluoride (PVDF) membrane, and reacted with primary antibodies, then followed by second antibody. The specific proteins were detected by the enhanced chemiluminescence detection system (ECL, Amersham). The equal loading of protein sample was verified with an actin-specific antibody.

### Flow cytometry

One million cells were incubated for 30 min on ice in staining medium (0.5% BSA in PBS) with the relevant antibodies and then washed with PBS prior to flow cytometric analysis of surface expression on MDSCs. For intracellular staining of IFN-γ, cells were labeled with anti-CD8-PE and anti-CD4-FITC, fixed, permeabilized in Cytofix/Cytoperm buffer (BD Biosciences) for 20 min at 4 °C, and washed with a 1 × Perm/Wash solution (BD Biosciences). The cells were then incubated with anti- IFN-γ -APC for 30 min on ice. After washing, the samples were analyzed using a LSRII (BD Pharmingen, San Diego, CA), and the results were analyzed using Flowjo 6.3.4 software (TreeStar).

### Apoptosis Analysis

Cells were treated with DMSO control or 20 μM curcumin or 10 ng/ml of DTX for 24 h at 37° and then harvested and stained for CD11b, Ly6C, Ly6G, CCR7 or Dectin-1 followed by Annexin V.

### Antisense clusterin expression

Raw264.7 cells or Ly6C^+^ MDSCs were transfected with either mouse clusterin siRNA or scramble control (final concentration 100 nM) using Lipofectamine 2000. Twenty-four hours after transfection, cells were treated with curcumin or DTX for 24 h prior to analysis of apoptosis. Cells were harvested and stained for 7AAD and Annexin APC using the Annexin V Apoptosis kit (BD Pharmingen).

### ROS production

The oxidation-sensitive dye, DCFDA, (Molecular Probes/Invitrogen) was used to measure ROS production by MDSC. Cells were incubated at room temperature in RPMI in the presence of 3 μM DCFDA for 30 min, washed with PBS, and then labeled with anti–CD11b-APC and anti–Gr1-PE antibodies. After incubation on ice for 20 min, cells were washed with PBS and analyzed using flow cytometry.

### Statistical analysis

Statistical analysis was performed using a 2-tailed Student’s *t* test and GraphPad Prism 5 software (GraphPad Software Inc.), with significance determined at *P* < 0.05. All data were expressed as the mean ± SE. For all experiments, the graphs represent the mean of three separate experiments and the error bars represent the standard error.

## Additional Information

**How to cite this article**: Zhou, J. *et al*. Therapeutic targeting of myeloid-derived suppressor cells involves a novel mechanism mediated by clusterin. *Sci. Rep*. **6**, 29521; doi: 10.1038/srep29521 (2016).

## Supplementary Material

Supplementary Information
