# Supplementary material for: Therapeutic targeting of myeloid-derived suppressor cells involves a novel mechanism mediated by clusterin
Source: Sci Rep. 2016 Jul 13;6:29521. doi: 10.1038/srep29521 (PMC4942787; doi:10.1038/srep29521)
Supplement: Supplementary Information [file srep29521-s1.doc]

**Therapeutic targeting of myeloid-derived suppressor cells involves a novel mechanism mediated by clusterin**

Junmin Zhou^1^, Sarah S Donatelli^1^, Danielle L Gilvary^1^, Melba Marie Tejera^1^, Erika A. Eksioglu^1^, Xianghong Chen^1^, Domenico Coppola^2^, Sheng Wei^1^, and Julie Y Djeu^1^

^1^H. Lee Moffitt Cancer Center, Department of Immunology

^2^H. Lee Moffitt Cancer Center, Department of Pathology

Corresponding Author: Julie Y. Djeu, Ph.D., H. Lee Moffitt Cancer Center, Department of Immunology, MRC 4072D, 12902 Magnolia Dr, Tampa, FL, U.S.A 33612, Phone: 813-745-3041, Fax: 813-745-7264; E-mail: [julie.djeu@moffitt.org](mailto:julie.djeu@moffitt.org).

**
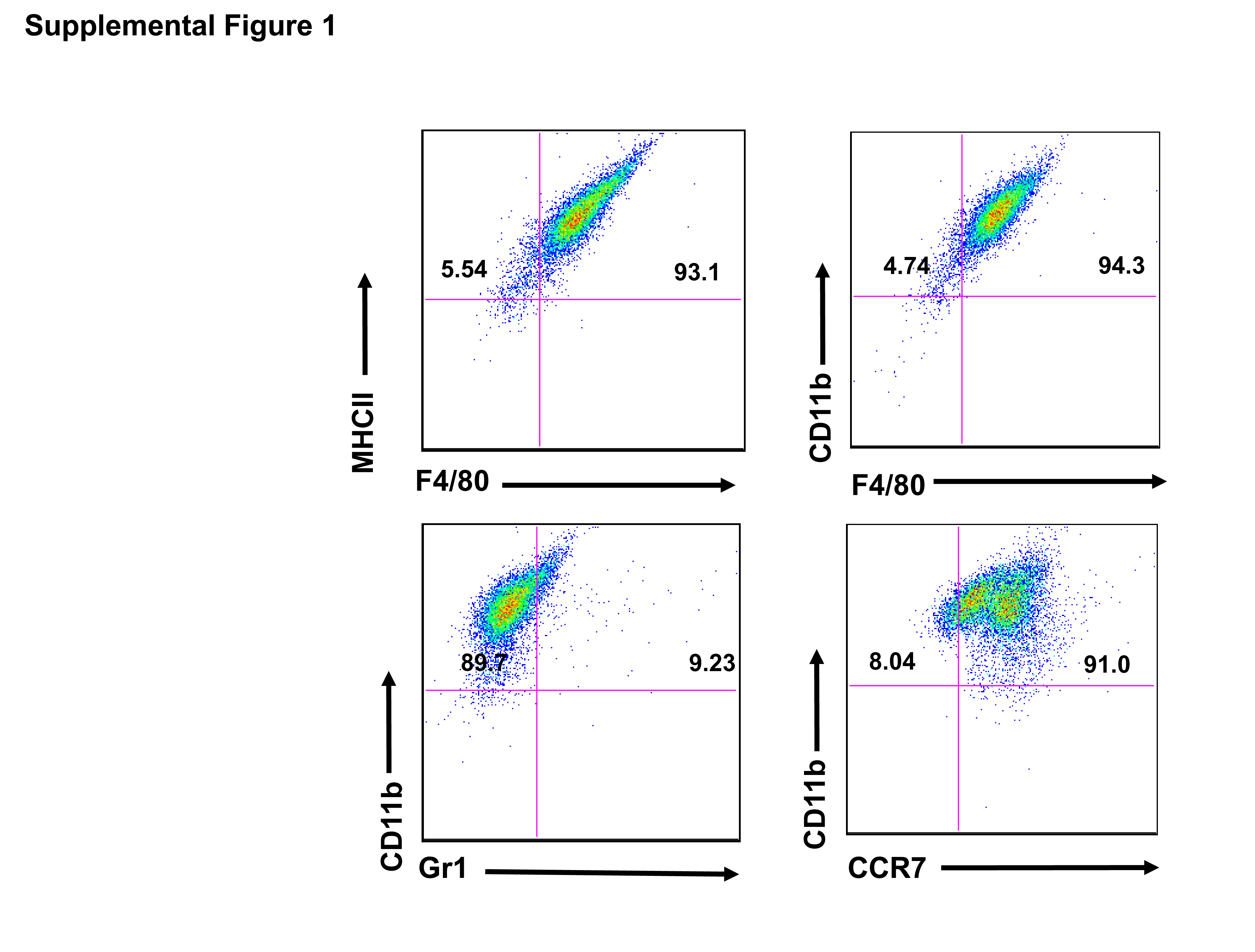
**

**Figure 1S. RAW267.4 cells have the phenotype of mature macrophages.**  Raw267.4 cells were dual stained with antibodies to CD11b and Gr1, F4/80 or CCR7 as well as antibodies to F4/80 with MHC-II for flow cytometric analysis of macrophage markers.
